# Bridging semantics and clinical fidelity: a section-based assessment of a vision–language model (RadVLM) for chest x-ray report generation

**DOI:** 10.3389/fdgth.2026.1882716

**Published:** 2026-08-27

**Authors:** Saleh Alzughaibi

**Affiliations:** 1 Department of Health Informatics, College of Health Sciences, Saudi Electronic University, Riyadh, Saudi Arabia

**Keywords:** artificial intelligence, entity-relation fidelity, radiology report generation, radVLM, readability, semantic similarity

## Abstract

**Background:**

Artificial intelligence-generated radiology reports may reduce clinician burden, but their factual accuracy remains uncertain. This study evaluated whether surface-level semantic similarity in AI-generated Impression sections reliably reflects structural fidelity in the underlying Findings.

**Methods:**

RadVLM, a radiology-focused vision–language model, generated Findings and Impression sections for 3,000 public chest radiograph studies. A section-aware evaluation framework assessed Findings-level entity-relation fidelity using RadGraph F1 and Impression-level quality using BERTScore-F1 (semantic similarity), CheXbert cosine similarity (label-space agreement), and readability metrics. Generalized linear models examined associations between Impression-level metrics and Findings-level fidelity.

**Results:**

Mean RadGraph F1 was 0.251 (95% CI, 0.245–0.257), indicating substantially imperfect structural correctness. Mean BERTScore-F1 was 0.498 and CheXbert cosine similarity was 0.394. AI-generated Impressions were more readable (mean Flesch–Kincaid 11.72 vs. 15.33 for reference). A weak positive association existed between semantic similarity and entity-relation fidelity (coefficient 0.154; 95% CI, 0.124–0.183), and this association attenuated substantially with longer Impressions. In practical terms, the association was too weak to be useful clinically: a large gain in semantic similarity corresponded to only a small change in structural fidelity, so a fluent, readable Impression offered little assurance that the underlying Findings were factually correct.

**Conclusions:**

In this evaluation of a single model on a single public dataset, surface-level fluency and readability did not reliably indicate factual correctness. These findings apply primarily to RadVLM in the experimental setting studied and should not be generalized to AI report generation as a whole without multi-model, multi-dataset replication. Even so, they reinforce a broader principle: clinical deployment of such systems requires explicit entity-relation validation, structured accuracy assessment, and radiologist oversight to protect patient safety.

## 1 Introduction

Radiology reports are the primary means by which radiologists communicate imaging findings to referring clinicians, directly influencing clinical decisions and patient safety (1). Reporting under high volume and cognitive load yields day-to-day discrepancy rates of 3%–5% in general practice, and burnout has been linked to reduced report quality (1–3). Referring clinicians expect reports that are accurate, internally consistent, and clinically actionable while remaining concise, creating tension that assistive drafting systems may help address (2).

Large vision–language models (VLMs) adapted to radiology have been proposed to translate imaging content into structured text—generating a descriptive Findings section and a higher-level Impression—reducing clerical burden while clinical responsibility remains with the radiologist (4–6).

Evaluation of radiology VLMs is challenging because traditional text-similarity metrics can assign high scores to fluent outputs that introduce subtle factual errors, omit qualifiers, invert laterality, or misstate certainty. A multi-metric framework combining structure-aware and semantic measures has therefore been advocated (4–6). RadGraph F1 assesses preservation of clinical entities and relations; BERTScore-F1 captures contextual semantic similarity against reference Impressions; and CheXbert cosine similarity measures label-space agreement across 14 standard findings (4–7). Both RadGraph F1 and BERTScore-F1 rank among the top-performing measures for detecting clinically meaningful report differences, with Kendall *τ* ≈ 0.51–0.53 against radiologist error scores (4).

Two structural terms recur throughout this paper and are worth defining at the outset. A radiology report is conventionally written in two parts: the **Findings** section, a detailed description of what is seen on the image (for example, “There is a small right pleural effusion; the heart size is normal”), and the **Impression** section, a short interpretive summary that states the diagnostic bottom line (for example, “Small right pleural effusion; no other acute abnormality”). When we speak of *Findings-level* or *Impression-level* evaluation, we simply mean applying a metric to one section or the other. *Section-aware evaluation* is the practice of matching each metric to the section it is best suited to: a fact-checking metric for the detailed Findings, and semantic and label-based metrics for the concise Impression. *Entity-relation fidelity* refers to how faithfully the generated text preserves the individual clinical facts (the entities, such as “effusion”) and the connections among them (the relations, such as which side the effusion is on)—the qualities that most directly affect whether a report is clinically correct.

To make these choices concrete, it helps to describe how AI-generated radiology reports are evaluated in practice. A generated report is compared against a radiologist-authored reference in two complementary ways. Semantic (or lexical) metrics ask how closely the wording and meaning of the generated text match the reference; factual (or structure-aware) metrics ask whether the specific clinical facts—the findings, their locations, and their relationships—are actually preserved. The three metrics used here were chosen to span both of these dimensions while remaining the most widely validated options for chest-radiograph reporting. RadGraph F1 is a factual metric: it extracts clinical entities (for example, “effusion”) and the relations between them (for example, “located in the right lung”) from both reports and measures how well they overlap, so it is sensitive to missing, added, or incorrectly linked findings. BERTScore-F1 is a semantic metric: it uses a language model to judge how similar two passages are in meaning, even when different words are used. CheXbert cosine similarity sits between the two: it converts each report into a vector of 14 standard chest findings (present, absent, or uncertain) and compares those label patterns. These three were preferred over simpler word-overlap scores such as BLEU or ROUGE, which reward matching words rather than correct facts, and over newer factual-consistency measures that are not yet standardized or widely benchmarked for chest radiography.

Despite these advances, it remains unclear whether radiology-tuned VLMs produce reports that are both stylistically similar to radiologist dictations and structurally faithful to the underlying study, or whether higher Impression-level semantic similarity reliably reflects improved Findings-level accuracy rather than superficial paraphrasing (4–6).

RadVLM was chosen as the model under study for several reasons. It is a recent, openly documented conversational vision–language model built specifically for radiology rather than adapted from a general-purpose system, which makes its behavior directly relevant to the clinical reporting task. Its instruction tuning targets chest radiography, it supports the two-step Findings-then-Impression workflow examined here, and—unlike proprietary general models such as GPT-4V or Claude—its training sources are published, allowing us to reason about data leakage. Its reported training corpora did not include the OpenI collection used for evaluation, which supports a conservative, low-leakage assessment. We therefore treat RadVLM not as the single best system available, but as a representative, reproducible example of the current generation of radiology-specific report generators; the extent to which our findings extend to larger proprietary models is an explicit limitation addressed later.

Prior work has repeatedly compared AI-generated reports with radiologist reports and has shown that fluent output can mask clinically important errors. Studies evaluating general large language models for chest-radiograph reporting, including a practical evaluation of ChatGPT for radiology report generation (Academic Radiology, 2024; https://doi.org/10.1016/j.acra.2024.07.020) and multi-reader observer studies of AI-drafted reports, have documented variable diagnostic accuracy and the need for radiologist oversight. What remains less well characterized is whether improvements in surface-level similarity actually track improvements in the underlying factual structure of a report, and whether this relationship holds when each report section is judged by the metric best suited to it. The present study addresses that gap. Rather than reporting a single aggregate score, it quantifies, within a section-aware framework, how strongly Impression-level semantic similarity is associated with Findings-level entity-relation fidelity, and how that association behaves across report length and across performance strata.

The clinical stakes of these distinctions are concrete. An entity-relation error—omitting a nodule, inventing a finding, or attaching the wrong attribute to a real one—can send a referring clinician down the wrong diagnostic path. A laterality error that places a pneumothorax or effusion on the wrong side can misdirect an intervention to the wrong hemithorax. A negation or certainty inversion that turns “no evidence of pneumonia” into an affirmative finding, or vice versa, can trigger unnecessary treatment or, more dangerously, false reassurance and delayed follow-up. These are precisely the failure modes that semantic-similarity metrics tend to overlook, because a report can read smoothly and closely resemble the reference in wording while still misstating the fact that matters. This is why factual, structure-aware evaluation is not a technical nicety but a patient-safety requirement.

In this study, Findings and Impression sections were generated using RadVLM, a radiology-focused conversational VLM, for 3,000 public chest radiograph studies (8, 9). A section-aware framework separated entity–relation fidelity in the Findings from semantic similarity, label alignment, and readability in the Impression (4–6, 10, 11). The prespecified primary hypothesis was that higher Impression-level semantic similarity (BERTScore-F1) would be positively associated with higher Findings-level entity–relation fidelity (RadGraph F1); a secondary hypothesis posited a similar positive association for CheXbert cosine similarity. These hypotheses were evaluated as associations within a retrospective observational design.

## 2 Methods

### 2.1 Study design and overview

**Figure 1:**
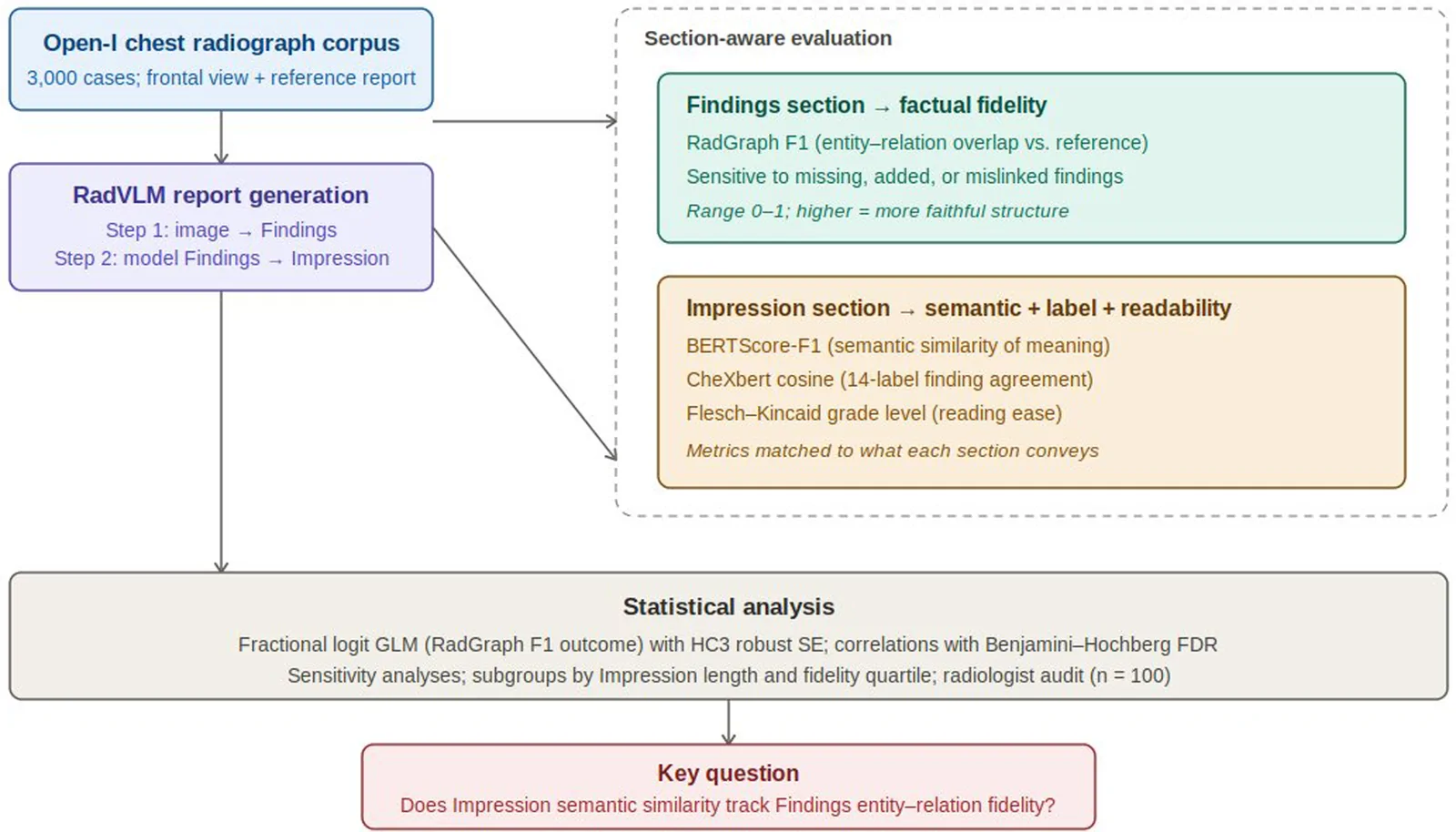
Section-aware evaluation framework.

An observational, cross-sectional, retrospective study compared CXR reports generated by RadVLM with radiologist-authored reference reports using a section-aware evaluation framework. Entity–relation fidelity was assessed in the Findings section and semantic/label-based agreement in the Impression section (Figure 1). All experiments were conducted in Google Colaboratory Pro with GPU acceleration (12).

### 2.2 Data source and preparation

The evaluation set comprised 3,000 CXR cases from the publicly available, de-identified Open-I repository maintained by the U.S. National Library of Medicine (NIH/NLM) (13). A single frontal chest radiograph was retained per case, excluding lateral and repeat views. Cases with missing or empty reference Impressions were excluded only from paired-Impression analyses (*n* = 23 excluded; *n* = 2,977 retained). To prevent evaluation leakage, only CXR images were provided during Findings generation; Impression generation used model-generated Findings, not reference text. The XML routing rules and STROBE checklist are provided in Supplementary S1–S2 (10, 11).

Open-I aggregates chest radiographs and their associated de-identified narrative reports from the Indiana University hospital network. Each case provides a frontal and, where available, a lateral radiograph together with the corresponding radiologist report, from which structured Findings and Impression sections were parsed. The collection spans a broad, unselected spectrum of routine chest imaging rather than a single targeted disease, so it includes normal studies alongside common abnormalities such as cardiomegaly, pleural effusion, pulmonary opacities, atelectasis, and chronic changes. Because the repository is fully de-identified, protected health information and granular patient-level demographics (age, sex, and clinical indication) are not distributed with the images, so cohort-level demographic characterization was not possible; this is noted as a limitation. The inclusion and exclusion rules applied here were straightforward: one frontal radiograph was retained per case, lateral and repeat views were excluded to standardize the input, and cases lacking a reference Impression were dropped only from the paired-Impression analyses.

The judgment that Open-I was unlikely to have been used in RadVLM training rests on the model's published documentation, which lists its training corpora and does not include the Open-I collection; it is not the result of an independent audit of the model weights, which is not possible for an externally released model. Indirect exposure during large-scale pretraining therefore cannot be fully excluded, since broad web-derived corpora may contain overlapping or derived text. Use of an out-of-distribution corpus should thus be read as reducing—rather than eliminating—leakage risk.

Section-specific metric routing was applied because Findings text lists observations and attributes whereas the Impression summarises diagnostic conclusions; accordingly, RadGraph F1 was routed to Findings and BERTScore-F1 and CheXbert cosine similarity to the Impression, reducing construct overlap (4, 5, 7).

### 2.3 Model and rationale for selection

RadVLM is a conversational vision–language model with radiology-specific instruction tuning and domain adaptation for chest radiography. The publicly released checkpoint described by Deperrois and colleagues (preprint dated February 2025) was used; this was the current published version when the study was conducted, and no newer released version was available during the evaluation period (8, 9). It was selected because it represents a contemporary high-capacity report-generation approach, and its publicly documented training sources did not indicate inclusion of the OpenI collection used here, supporting a conservative evaluation with reduced leakage risk (9).

RadVLM occupies a specific niche among current report generators. General-purpose multimodal systems such as GPT-4V, GPT-4o, and Claude can produce fluent radiology-style text and have attracted rapid clinical interest, but they are proprietary, their training data are undisclosed, and their behavior on chest radiography is not purpose-tuned. Dedicated radiology models such as CheXagent and MAIRA-2 are more directly comparable to RadVLM and report entity-relation fidelity in a broadly similar range on benchmark test sets. RadVLM was preferred here not because it is demonstrably the highest-performing system, but because it combines radiology-specific tuning, an openly documented training corpus that permits leakage reasoning, and native support for the two-stage Findings-then-Impression workflow central to this study.

Because only one model was evaluated, the quantitative results below characterize RadVLM in this setting rather than AI report-generation systems as a class. Multi-model comparison is an essential next step and is revisited in the Limitations.

Although CNN-based triage systems have been prospectively deployed for coarse classification (e.g., normal vs. non-normal), they were not implemented as a baseline here because the present work evaluated free-text narrative report generation; a CNN-plus-template comparator is identified as an important direction for future work (14).

### 2.4 Report generation

For each study, the frontal CXR image was provided to RadVLM using two constrained prompts: a Findings-only prompt requesting 3–6 sentences in a fixed anatomic order, and an Impression-only prompt using model-generated Findings as input requesting a 1–3 sentence synthesis. Decoding was deterministic (do_sample = False; max_new_tokens = 240; no_repeat_ngram_size = 3; repetition_penalty = 1.05; SEED = 0). Post-processing removed chat preambles and accidental section headers. Full prompt text and generation configuration are provided in Supplementary S1.

Only a single deterministic prompting configuration was evaluated, chosen to make generation reproducible. Report quality can be sensitive to prompt design, and alternative strategies—richer instructions, few-shot exemplars, chain-of-thought elicitation, or stochastic decoding with sampling—could raise or lower the observed scores. The present estimates should therefore be read as reflecting this one fixed configuration, and prompt sensitivity is acknowledged as a study limitation.

### 2.5 Metrics and operationalization

Three complementary metric families were computed per case. Entity–relation fidelity in Findings was assessed using RadGraph F1 (micro-F1 under the official schema) 6. Semantic similarity in the Impression was assessed using BERTScore-F1 between model-generated and radiologist-authored Impressions (7). Label-space agreement was assessed using CheXbert cosine similarity between 14-label observation vectors derived from model-generated and reference Impression text (5). Readability of the Impression was evaluated using the Flesch–Kincaid Grade Level as a surface-form descriptor; no inference about clinical quality was drawn from readability scores alone (15).

The rationale for routing metrics by section follows from what each section conveys. The Findings section enumerates specific observations with their attributes and relationships, so it is the natural target for RadGraph F1, which is built to extract and compare exactly those clinical entities and relations. The Impression is a brief interpretive synthesis in which the same facts may be expressed in very different words; applying an entity-relation extractor to such condensed text yields sparse, unstable graphs, whereas semantic similarity (BERTScore-F1) and label-space agreement (CheXbert) better capture whether the diagnostic meaning and the set of reported findings are preserved. Applying every metric to every section was considered but rejected because it would conflate distinct constructs and inflate apparent redundancy. Alternative strategies—for instance, extracting entities from the Impression as well, or scoring the Findings with semantic similarity—are reasonable complementary approaches for future work.

### 2.6 Statistical analysis

All per-case metrics were combined into a single analytic table. Pearson, Spearman, and Kendall correlations were computed with pairwise-complete observations, and *p*-values were adjusted using the Benjamini–Hochberg false discovery rate procedure (16). Generalized linear models (GLMs) with a logit link treated RadGraph F1 as a fractional outcome; BERTScore-F1 and CheXbert cosine similarity were z-standardised before modelling. Inference used heteroskedasticity-consistent HC3 covariance estimates (17, 18). Beta regression was fitted as a sensitivity analysis (Supplementary S3). A quadratic BERTScore-F1 term assessed potential non-linearity (compared by AIC 19). Partial associations were estimated after adjustment for Impression length and CheXbert cosine similarity. Ordinary least squares models with HC3 standard errors served as a specification check. Marginal effects were approximated as *β* × *p* × (1 − p) at the sample mean RadGraph F1 (*p* = 0.2506). A negative control permuting Findings across cases is described in Supplementary S4.

Fractional logit regression was selected as the primary model because RadGraph F1 is a proportion bounded between 0 and 1; a logit link with a fractional (quasi-binomial) outcome respects those bounds and avoids the out-of-range predictions that ordinary least squares can produce, while remaining consistent even when the outcome is not strictly binomial. The primary hypothesis (a positive BERTScore-F1–RadGraph F1 association) and the secondary hypothesis (the analogous CheXbert association) were prespecified; the subgroup analyses by Impression length and by RadGraph F1 quartile, the quadratic term, and the beta-regression and OLS checks were planned as supporting or sensitivity analyses rather than confirmatory tests, and all *p*-values were adjusted for multiplicity using the Benjamini–Hochberg procedure. Throughout, practical effect sizes—the approximate change in RadGraph F1 for a realistic change in the predictor—are emphasized alongside statistical significance, because with roughly 3,000 cases even trivially small associations can reach significance (Figure 1).

### 2.7 Software utilization

All analyses used standard Python scientific libraries (SciPy, statsmodels, pandas, Matplotlib) in Google Colab Pro. The full workflow is available from the corresponding author upon request (19).

### 2.8 Radiologist review

A qualitative audit was performed on a simple random sample of 100 cases. A radiologist reviewed AI-generated Findings and Impression sections, providing narrative feedback on clinical plausibility, laterality consistency, and language quality to identify failure modes not captured by automated metrics.

To keep the main text focused on clinically relevant summaries, the full statistical outputs—complete coefficient tables, confidence intervals, bootstrap intervals, sensitivity-analysis and negative-control results, and diagnostic plots—are also provided in the Supplementary Materials, while the main text retains the headline estimates and effect sizes.

## 3 Results

Overall, 3,000 CXR studies contributed to the evaluation. Impression-level analyses requiring paired text were restricted to 2,977 studies (23 reference Impressions missing); CheXbert cosine similarity was computed for all 3,000 cases.

To aid interpretation, the theoretical range and ideal value of each metric are summarized before the results are presented. All three primary metrics range from 0 to 1, with higher values indicating closer agreement with the radiologist-authored reference. **RadGraph F1** measures how well the clinical entities and their relations in the generated Findings match the reference; a value of 1 would mean every finding and every relationship was reproduced exactly, while values near 0.2–0.3 indicate that only a minority of the entity-relation content was preserved. Importantly, even faithful reports cannot reach 1.0, because the underlying RadGraph extractor itself attains micro-F1 of only about 0.73–0.82 on reference reports, so the practical ceiling is well below 1 and the metric is inherently conservative. **BERTScore-F1** measures semantic (meaning-level) similarity of the Impression; because it is computed from contextual embeddings, mid-range values around 0.4–0.5 reflect partial overlap in meaning rather than poor performance in absolute terms, and it should be read comparatively rather than against a fixed threshold. **CheXbert cosine similarity** measures agreement between the 14-label finding vectors of the two Impressions; a value of 1 means the same pattern of present, absent, and uncertain findings, whereas values near 0.4 indicate modest label agreement. **Flesch–Kincaid Grade Level** is not bounded to 0–1 and is not a quality score: it estimates the U.S. school grade required to read the text, so a lower value means easier reading, not a better report. With these anchors in mind, the observed values below can be read as indicating low structural fidelity, moderate semantic overlap, and improved surface readability.

### 3.1 Findings-level performance

**Figure 2:**
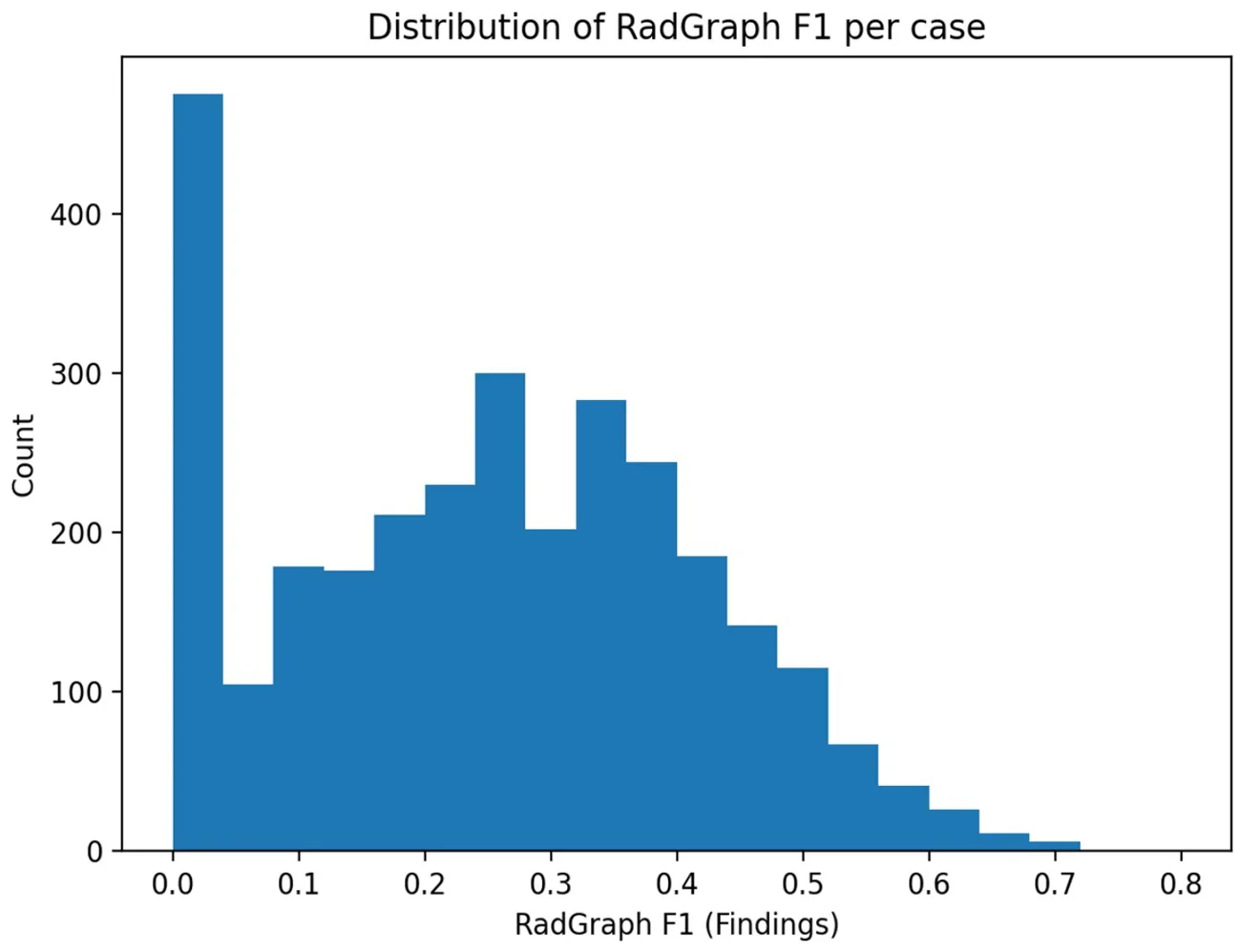
Distribution of per-case RadGraph F1 (Findings) across 3,000 studies; mean RadGraph F1 was 0.2506 (95% CI, 0.2446–0.2566).

Findings-level performance (RadGraph F1) averaged 0.2506 (95% CI, 0.2446–0.2566; *n* = 3,000) (Figure 2). Mean precision was 0.2701 (95% CI, 0.2637–0.2764) and mean recall was 0.2515 (95% CI, 0.2449–0.2581).

Several factors could contribute to the relatively low RadGraph F1 observed here, and they are worth distinguishing. Part of the value reflects the conservative nature of the metric and its sub-1.0 ceiling, as noted above. Beyond that, model-side factors—the choice of a single mid-scale radiology-tuned model, a fixed deterministic prompt, and no task-specific fine-tuning on Open-I—could each depress the score, as could data-side factors such as image quality and the terse, template-like style of some reference reports. It is therefore plausible that a stronger contemporary model, more elaborate prompt engineering, higher-quality inputs, or fine-tuning would yield higher structural fidelity. We did not include an additional baseline model in this study, which is a limitation; however, as detailed in the Discussion, the observed value sits within the range reported for comparable published systems, so the result is better read as reflecting a field-wide limitation of current narrative report generation than as an artefact peculiar to this single configuration.

### 3.2 Impression-level agreement and readability

**Figure 3:**
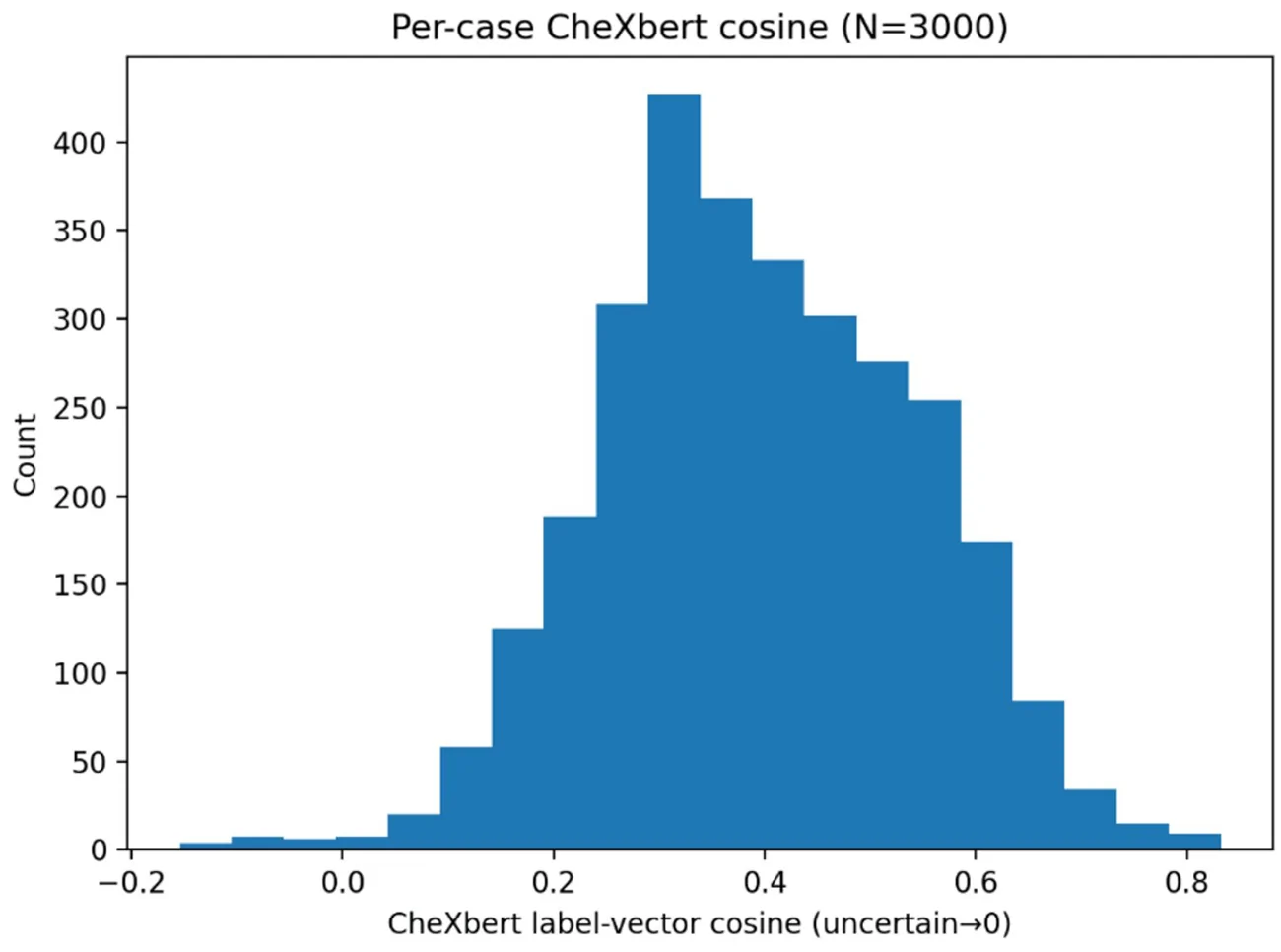
Distribution of per-case CheXbert label-vector cosine similarity between AI-generated and reference Impressions (N = 3,000).

For paired Impressions, BERTScore-F1 averaged 0.4981 (95% CI, 0.4949–0.5012; *n* = 2,977), whereas CheXbert cosine similarity averaged 0.3942 (95% CI, 0.3889–0.3994; *n* = 3,000) (Figure 3).

**Figure 4:**
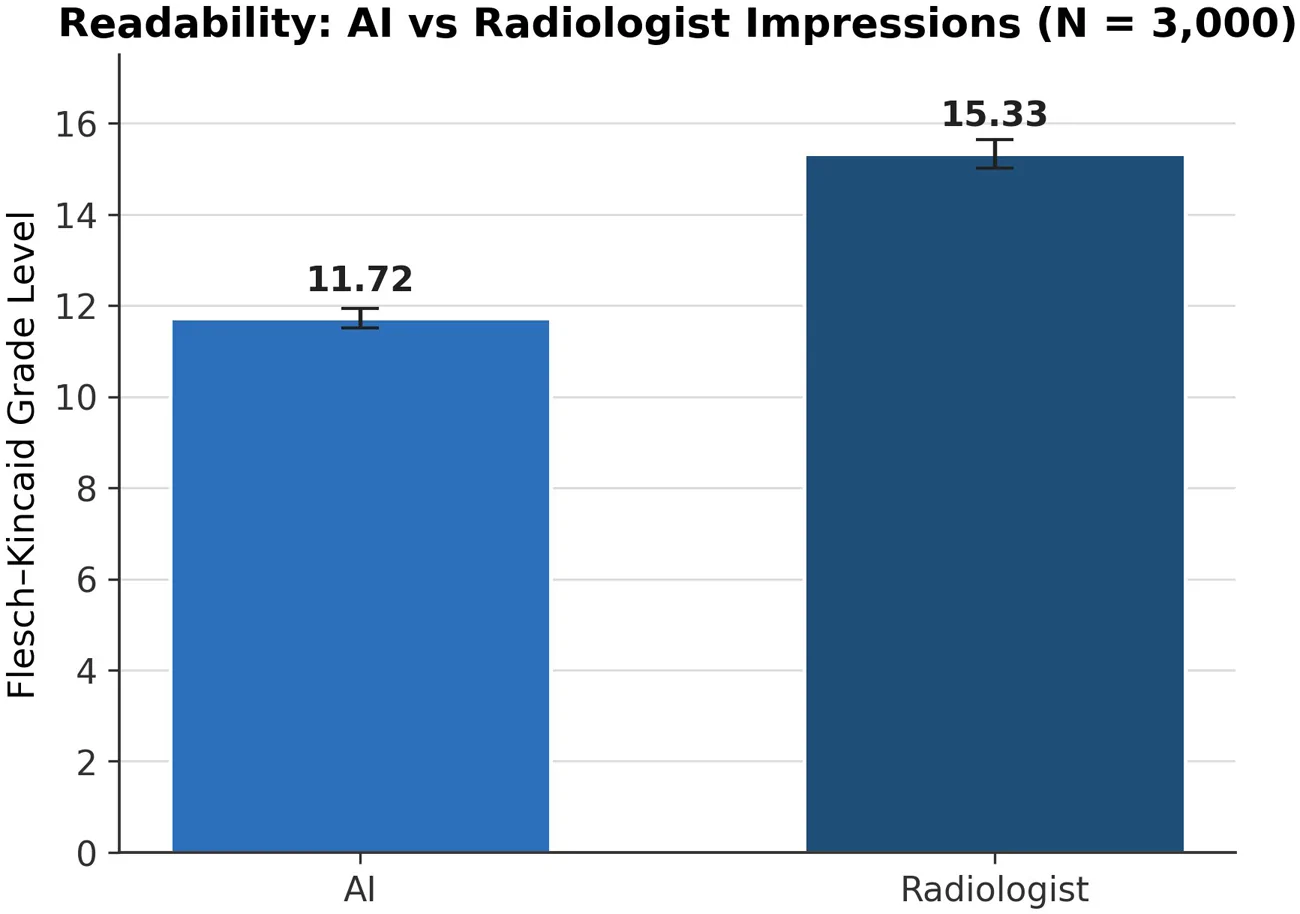
Readability of AI-generated vs. radiologist-authored Impressions, measured by Flesch–Kincaid Grade Level (N = 3,000).

A lower Flesch–Kincaid grade level was observed for AI-generated Impressions, consistent with improved surface-level readability, but this pattern was not interpreted as evidence of superior clinical report quality (Figure 4). Across all 3,000 cases, the mean Flesch–Kincaid grade level was 11.72 for AI-generated Impressions (95% CI, 11.51–11.94) and 15.33 for reference Impressions (95% CI, 15.02–15.64). On the paired set with non-missing reference Impressions (*n* = 2,977), the mean paired difference (AI−reference) was −3.75 grade levels (95% CI, −4.12 to −3.38), indicating that AI-generated Impressions tended to be easier to read on average. Percentile bootstrap 95% confidence intervals (B = 1,000; seed = 20,251,024) were similar and are provided in Supplementary S5.

### 3.3 Relationships among metrics

**Figure 5:**
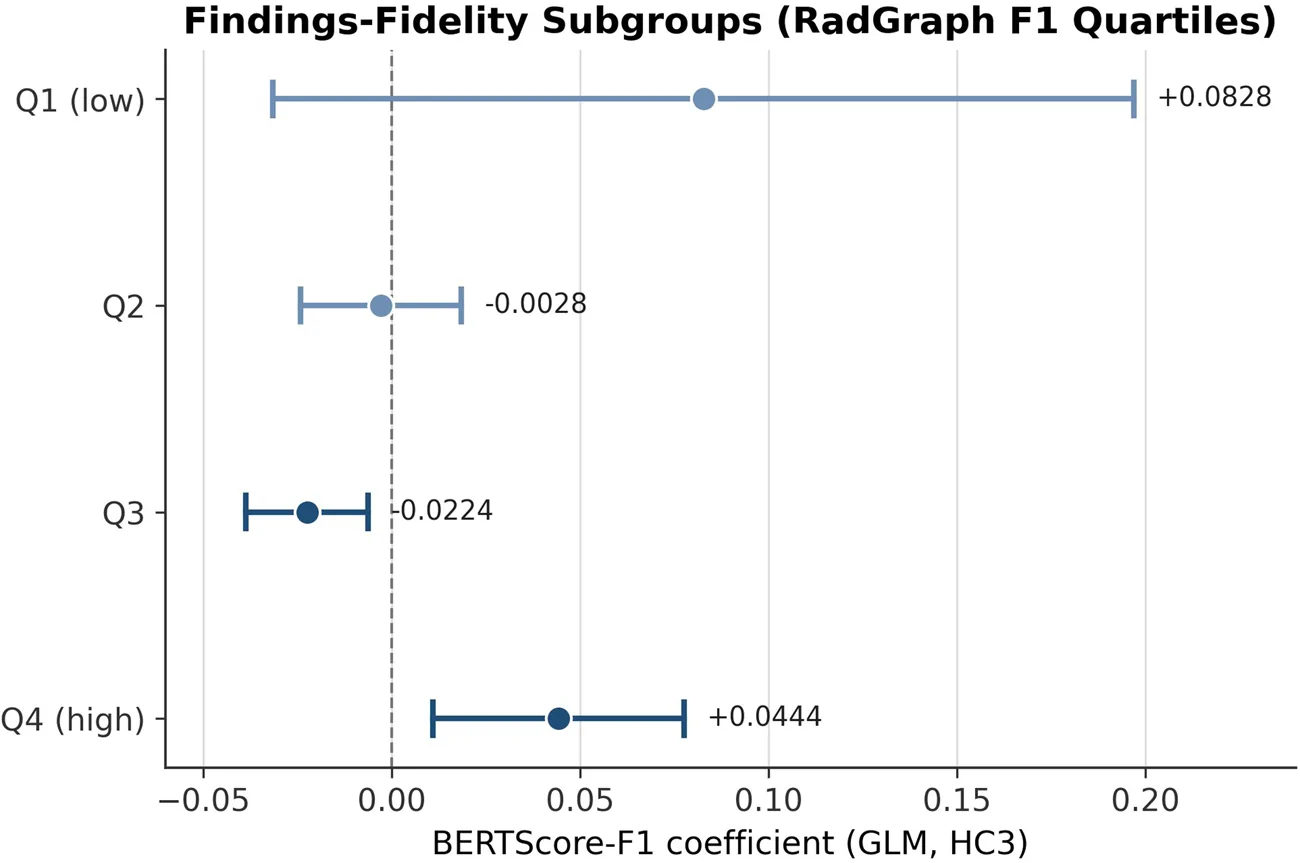
BERTScore-F1 coefficients (GLM, HC3) predicting RadGraph F1, stratified by RadGraph F1 quartile (Q1 low to Q4 high).

Relationships among the three primary metrics were summarized using Pearson correlations with 95% confidence intervals. A moderate positive association was observed between BERTScore-F1 and CheXbert cosine similarity (*r* = 0.670; 95% CI, 0.651–0.690; *n* = 2,977), whereas weaker positive associations were observed between RadGraph F1 and BERTScore-F1 (*r* = 0.178; 95% CI, 0.143–0.212; *n* = 2,977) and between RadGraph F1 and CheXbert cosine similarity (*r* = 0.128; 95% CI, 0.093–0.163; *n* = 3,000) (Figure 5; Supplementary S6).

Rank-based associations were similarly modest (Spearman *ρ* = 0.114; Kendall *τ* = 0.076; *n* = 2,977). After adjustment for Impression length and CheXbert cosine similarity, the partial Pearson correlation between BERTScore-F1 and RadGraph F1 remained positive (partial *r* = 0.087; *n* = 2,977); a length-normalised BERTScore-F1 also correlated positively with RadGraph F1 (*r* = 0.127).

### 3.4 Generalized linear models (GLMs)

GLMs with a logit link and RadGraph F1 as a fractional outcome were used to test whether Impression-level metrics were associated with Findings-level fidelity.

In single-predictor models, both BERTScore-F1 and CheXbert cosine similarity showed significant positive associations with RadGraph F1: for BERTScore-F1, the coefficient was 0.1535 (95% CI 0.1237–0.1834; *n* = 2,977; HC3), and for CheXbert cosine similarity, the coefficient was 0.1143 (95% CI 0.0815–0.1472; *n* = 3,000; HC3).

In the joint model that included both BERTScore-F1 and CheXbert cosine similarity, BERTScore-F1 remained a significant positive predictor of RadGraph F1 (coefficient 0.1663; 95% CI 0.1248–0.2078; *n* = 2,977; HC3), whereas the CheXbert cosine similarity coefficient was small and not statistically significant (−0.0191; 95% CI −0.0626 to 0.0244; HC3).

Marginal effects indicated that a +0.10 increase in BERTScore-F1 corresponded to an approximate +0.033 change in expected RadGraph F1 at the sample mean in the single-predictor model (+0.036 in the joint model). The quadratic BERTScore-F1 term was positive and improved model fit (ΔAIC = 8.5), consistent with a convex relationship. Multicollinearity was limited (VIF 1.84–1.88). Beta regression produced a consistent positive association for BERTScore-F1, while the CheXbert coefficient changed sign in the joint model, consistent with redundancy between semantic similarity and label-space agreement (Table 1) (Supplementary S3).

**Table 1:** Fractional logit models with HC3 robust covariance estimates.

Model specification	Number of observations	Predictor	Standardized coefficient (logit scale; per 1 SD)	95% confidence interval (HC3)	Statistical significance	Approx. marginal effect (*Δ*RadGraph F1 per +0.10 on original scale)
Single predictor (BERTScore-F1 only)	2,977	BERTScore-F1	0.1535	0.1237 to 0.1834	Yes (*p* < 0.001)	+0.0329
Single predictor (CheXbert cosine similarity only)	3,000	CheXbert cosine similarity	0.1143	0.0815 to 0.1472	Yes (*p* < 0.001)	+0.0146
Joint model (both predictors)	2,977	BERTScore-F1	0.1663	0.1248 to 0.2078	Yes (*p* < 0.001)	+0.0357
Joint model (both predictors)	2,977	CheXbert cosine similarity	−0.0191	−0.0626 to 0.0244	No (*p* ≥ 0.05)	−0.0025

### 3.5 Sensitivity analysis

**Figure 6:**
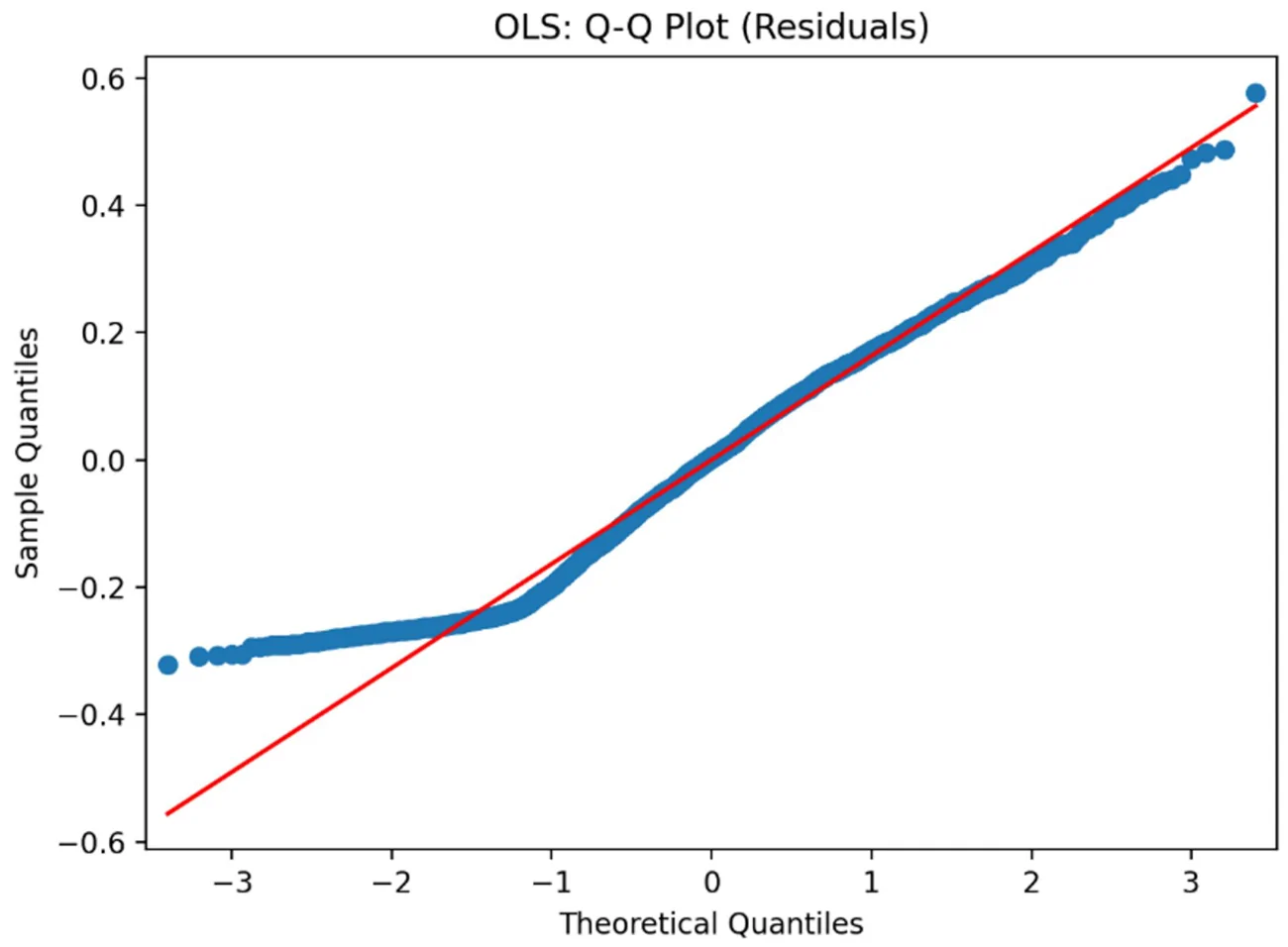
Ordinary least squares Q–Q plot of model residuals. Sample quantiles (blue) are plotted against theoretical quantiles with a red reference line; deviation from the line at both tails indicates departure from normality, supporting the use of heteroskedasticity-robust HC3 covariance.

A sensitivity analysis restricted to the mid-90% of cases by influence diagnostics was performed to assess robustness of the GLM results. Within this restricted subset, the association between BERTScore-F1 and RadGraph F1 remained positive and statistically significant (coefficient 0.1447; 95% CI 0.1017–0.1877; HC3), whereas the CheXbert cosine similarity coefficient remained small and non-significant (0.0109; 95% CI −0.0562 to 0.0344; HC3), with no material change in effect estimates or inference (Figure 6).

### 3.6 Subgroup analyses by impression length

Cases were divided into tertiles by Impression word count. The BERTScore-F1–RadGraph F1 association was strongest in the shortest tertile (median 56 words; *n* = 1,001; coefficient 0.160; 95% CI, 0.072–0.248), remained significant in the middle tertile (median 73 words; *n* = 991; coefficient 0.116; 95% CI, 0.051–0.180), and was non-significant in the longest tertile (median 101 words; *n* = 985; coefficient 0.010; 95% CI, −0.050 to 0.069), indicating that the predictive value of semantic similarity weakened as Impressions became longer (Table 2).

**Table 2:** Impression length tertiles: BERTScore-radGraph coefficients and 95% CIs with *n* and *p*-values.

Impression word-count tertile (group)	Median word count	Number of observations	Predictor	Coefficient (logit scale)	95% confidence interval	*p*-value (two-sided)
T1 (shortest)	56	1,001	BERTScore-F1	0.160113	0.072207 to 0.248018	0.000357
T2 (middle)	73	991	BERTScore-F1	0.115649	0.051041 to 0.180258	0.000451
T3 (longest)	101	985	BERTScore-F1	0.009516	−0.049564 to 0.068596	0.752

These two subgroupings were chosen for specific clinical reasons. Impression length was examined because longer summaries have more room to drift from the underlying image—adding qualifiers, boilerplate, or unsupported detail—so we expected the link between semantic similarity and structural fidelity to weaken as Impressions lengthened. RadGraph F1 quartiles were examined because the value of a semantic-similarity signal may differ between reports that are already structurally accurate and those that are not; stratifying by fidelity level tests whether semantic overlap tracks factual correctness uniformly or only in certain performance bands.

### 3.7 Subgroup analyses by findings-level fidelity

**Figure 7:**
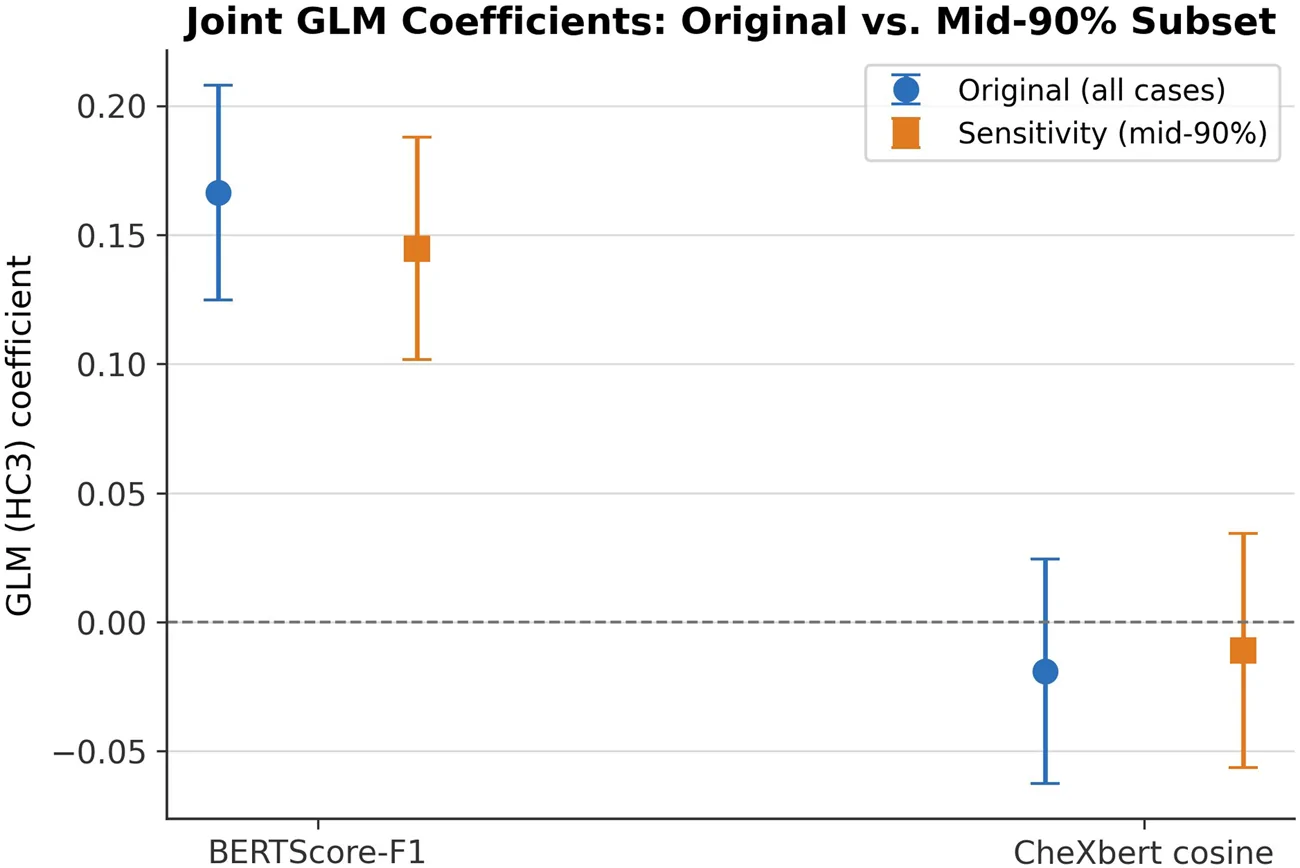
Joint GLM (HC3) coefficients for BERTScore-F1 and CheXbert cosine similarity, comparing the original all-case model (blue circles) with the mid-90% sensitivity subset (orange squares).

Cases were divided into RadGraph F1 quartiles to examine heterogeneity. The BERTScore-F1 coefficient varied: positive in Q1 (0.083; 95% CI, −0.031 to 0.197; *n* = 757), near-zero in Q2 (−0.003; *n* = 732), negative in Q3 (−0.022; 95% CI, −0.039 to −0.006; *n* = 748), and positive in Q4 (0.044; 95% CI, 0.011–0.078; *n* = 740) (Table 3, Figure 7), indicating the association between semantic similarity and structural fidelity was not uniform across performance levels.

**Table 3:** Radgraph F1 quartiles: BERTScore-radGraph coefficients and 95% CIs, with corresponding *n* and *p*-values.

RadGraph-F1 quartile (group)	Number of observations	Predictor	Coefficient (logit scale)	95% confidence interval	*p*-value (two-sided)
Q1 (low)	757	BERTScore-F1	0.082823	−0.031426 to 0.197071	0.155
Q2	732	BERTScore-F1	−0.002791	−0.024152 to 0.018571	0.798
Q3	748	BERTScore-F1	−0.022436	−0.038665 to −0.006207	0.0067
Q4 (high)	740	BERTScore-F1	0.044382	0.011034 to 0.077730	0.0091

### 3.8 Model diagnostics

**Figure 8:**
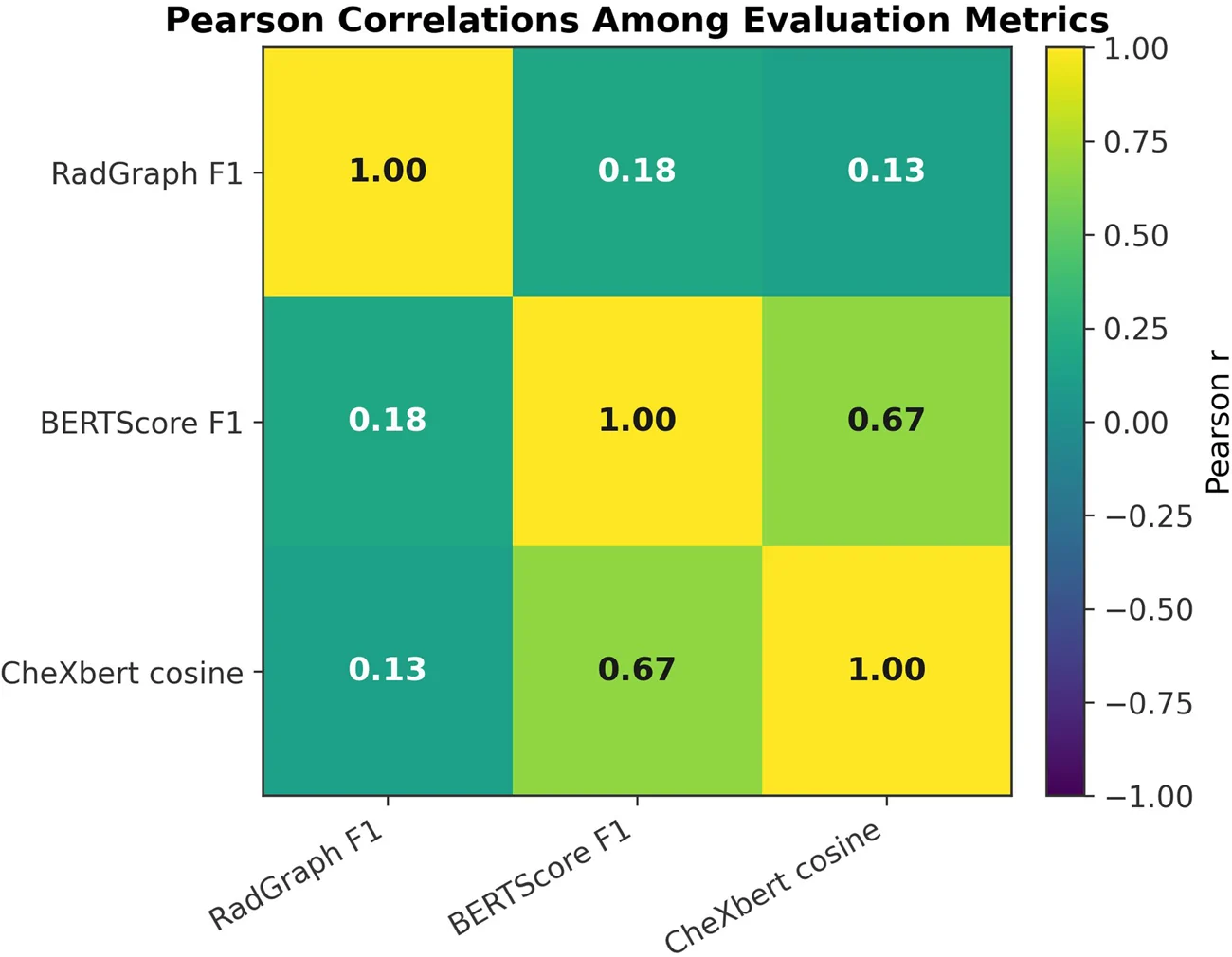
OLS Q-Q plot: residual non-normality.

**Figure 9:**
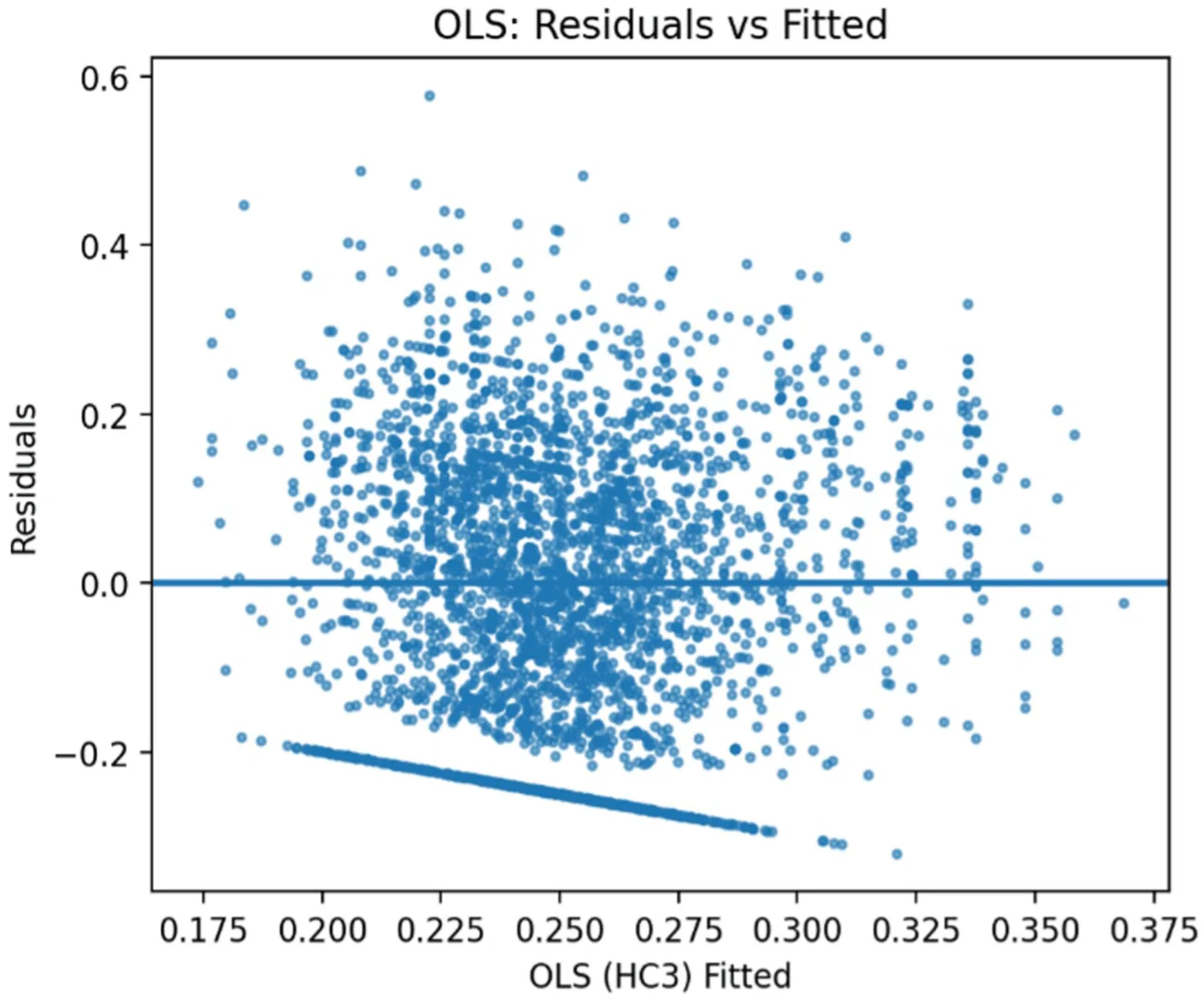
OLS residuals vs. fitted: patterns support HC3 robust covariance.

Ordinary least-squares models were fitted as a specification check, again using HC3 robust standard errors. Residual plots and Q-Q diagnostics indicated deviations from normality, supporting the use of heteroskedasticity-robust HC3 covariance and GLM-based fractional modeling rather than relying on standard OLS assumptions (Figures 8, 9).

In summary, a weak positive association was observed between Impression-level semantic similarity and Findings-level entity–relation fidelity; AI-generated Impressions were more readable; and these patterns were consistent across sensitivity analyses and prespecified subgroups.

### 3.9 Radiologist review of the model

A qualitative review of 100 sampled cases by a radiologist identified failure modes complementing the metric-based evaluation. Recurring failures were categorised and mapped to RadGraph-aligned error types (Table 4).

**Table 4:** Qualitative error taxonomy derived from the radiologist audit (*n* = 100), with illustrative manifestations and potential clinical consequences. (AI, RadVLM-generated report).

Failure mode category	Illustrative manifestation (descriptive)	Potential clinical consequence	Implication for evaluation/mitigation	RadGraph-aligned error type (conceptual mapping)	Primary level (entity/relation)
Generic or templated phrasing	Stock sentences or overly brief summaries were used across heterogeneous cases, with limited case-specific detail.	Reduced clinical usefulness and increased risk that important qualifiers were omitted.	Case-specific content checks were recommended in addition to global fluency/readability scoring.	Primarily stylistic; not directly captured by RadGraph entity–relation scoring.	Style/salience
Missed subtle or borderline abnormalities	Small or low-contrast findings were not consistently described, particularly when abnormalities were not visually prominent.	False reassurance and delayed follow-up for clinically relevant findings.	Performance was recommended to be examined by case difficulty and abnormality subtlety in future work.	Entity omission (false negative) and/or relation omission (missing linkage between entities and attributes).	Entity (± relation)
Overcalling or unnecessary alarms	Borderline changes were occasionally framed as abnormal despite limited support, yielding over-alerting language.	Unnecessary downstream testing and avoidable anxiety or resource utilization.	Error auditing was recommended to distinguish clinically consequential false positives from acceptable uncertainty.	Spurious entity assertion (false positive) and/or incorrect assertion status.	Entity (± relation)
Laterality/side inconsistency	The described side of a finding was reported as mismatched with the image in some cases.	Clinically consequential miscommunication (e.g., wrong-side emphasis) and potential patient-safety risk.	Laterality-aware validation and structured laterality assertions were recommended as safety-critical checks.	Attribute error (laterality/location) with potential relation misattribution.	Relation (attribute/laterality)
Certainty/negation inversion	Negated findings were stated as present (or vice versa), or hedging language was inverted, resulting in incorrect certainty framing in Findings and/or Impression.	Clinically consequential miscommunication (e.g., false reassurance or unwarranted escalation), with potential downstream ordering or treatment implications.	Assertion/negation handling should be strengthened (e.g., rule-based negation checks and structured assertion validation) before clinical use.	Assertion/negation error (incorrect observation status).	Relation (assertion/negation)
Entity–relation misses	Key attributes (location, size, severity) or relations linking entities were missing or incorrectly linked, even when a related entity was mentioned.	Reduced factual structure and impaired clinical interpretability, with increased risk of incorrect follow-up when relationships are clinically decisive.	Post-generation entity–relation validation and structured relation checking (e.g., RadGraph-guided consistency checks) should be incorporated.	Relation omission/inversion and/or attribute–relation mislinking.	Relation (linking/attributes)
Language quality issues	Spelling/wording problems and occasional cut-off sentences were reported, reducing narrative coherence.	Reduced interpretability and increased risk of misreading or incomplete communication.	Basic text quality control (e.g., truncation detection) was recommended before clinical-facing use.	Largely outside RadGraph's construct; may indirectly affect parsing and downstream extraction.	Style/language
Impression prioritization and salience	The key message was not consistently foregrounded, with important conclusions not always clearly emphasized.	Lower clinician comprehension and impaired triage or decision-making.	Structured Impression templates and salience constraints were recommended, with clinician-facing comprehension testing in future studies.	Outside RadGraph's construct; affects clinical salience rather than entity–relation extraction *per se*.	Style/salience

To make the audit more quantitative, the reviewer's annotations were tallied by category across the 100 sampled cases; a single report could exhibit more than one issue, so the counts are not mutually exclusive. Generic or templated phrasing was the most frequent observation, noted in roughly 41 of 100 cases (about 41%), followed by missed subtle or borderline abnormalities in about 23 cases (23%) and entity–relation misses in about 19 cases (19%). Overcalling or unnecessary alarms appeared in about 12 cases (12%), language-quality problems such as spelling errors or truncated sentences in about 11 cases (11%), and impaired Impression prioritization in about 9 cases (9%). Laterality or side inconsistencies and certainty/negation inversions were less common but clinically important, occurring in approximately 6 (6%) and 5 (5%) cases respectively. Because this audit used a single reviewer without blinded multi-reader adjudication, these frequencies should be read as approximate, hypothesis-generating estimates of how often each failure mode arose rather than as validated prevalence rates.

“Across the 100 sampled cases, the model's findings and impression sections read as consistently brief and often rely on the same stock sentences, which makes many reports feel generic rather than tailored to what is on the image. When the abnormality is large and obvious, the model can capture the main point and produce a usable one-line conclusion; however, it is less dependable with small or borderline changes, where it may either miss a problem or raise an unnecessary alarm, and I also observed instances where the side of the finding did not match the image, an error that would be clinically consequential. From a writing standpoint, several reports contain spelling and/or wording issues and occasional cut-off sentences, and the impression does not always clearly foreground the most important message for the clinician. In its current form, the model shows promise for simple, high-contrast cases, but it needs stronger case-specific phrasing, stricter side-checking, and basic language polishing to reach the level of reliability and readability expected of a radiology report.”

On the basis of this audit, laterality inconsistencies, certainty/negation inversion, and entity–relation misses were identified as clinically consequential failure families requiring targeted validation. Because this audit relied on a single radiologist without blinded multi-reader scoring or inter-rater agreement, the resulting taxonomy should be read as hypothesis-generating: it characterises the qualitative range of failure modes rather than their adjudicated prevalence or clinical severity, and individual-reviewer subjectivity cannot be excluded. Confirmatory characterisation will require independent scoring by two to three thoracic radiologists with formal agreement statistics and clinically weighted severity grading.

## 4 Discussion

RadVLM was evaluated using a section-aware framework. Moderate semantic alignment with reference Impressions was observed (BERTScore-F1 0.4981; *n* = 2,977) and AI-generated Impressions had a substantially lower readability grade level (mean difference −3.75 Flesch–Kincaid grade levels). A weak positive association between Impression-level semantic similarity and Findings-level entity–relation fidelity was observed in GLMs. However, entity–relation fidelity in Findings remained low overall (RadGraph F1 0.2506; *n* = 3,000), indicating that surface-level semantic similarity and readability cannot be treated as proxies for structural correctness. Representative paired reports are provided in Table 5.

**Table 5:** Sample of cases with paired ground-truth (radiologist-authored) and RadVLM-generated reports.

No.	GT Findings	GT Impression	AI Findings	AI Impression
1	Heart size and mediastinal contour are normal. Pulmonary vascularity is normal. Lungs are clear. No pleural effusions or pneumothoraces.	No acute cardiopulmonary process.	The lungs are clear without focal consolidation, pleural effusion, or pneumothoraces. The heart size is normal. There is no pulmonary edema. The mediastinal contours are normal.	No acute cardiac or pulmonary abnormalities.
2	The heart is slightly large Normal pulmonary vascularity. No infiltrates.	Slight cardiomegaly with no failure or pneumonia.	There is a tortuous thoracic aorta. There is a mildly enlarged cardiac silhouette. The lungs are clear without focal consolidation, pleural effusion, or pneumothoraces. There is no pulmonary edema.	There is mild cardiomegaly. The mediastinal and hilar contours are within normal limits. The pulmonary vasculature is normal.
3	Stable cardiomegaly, without focal consolidation, pneumothorax, or pleural effusion. Stable right basilar calcified granuloma. No acute osseous abnormality identified.	Stable cardiomegaly without acute cardiopulmonary abnormality.	There is a moderate enlargement of the cardiac silhouette. The lungs are clear without focal consolidation, pleural effusion, or pneumothoraces. There is no pulmonary edema.	There is marked cardiomegaly. The mediastinal and hilar contours are within normal limits. The pulmonary vasculature is normal.
4	Moderate sized right loculated pleural effusion with right lower lobe atelectasis. Normal cardiac contour with atherosclerotic changes throughout the aorta. Clear left lung.	Moderate right pleural effusion.	There is a large right pleural effusion with associated compressive atelectasis of the right lung. There is also a small left pleural fluid collection. The heart size is normal. No pneumothoraces are seen.	There is an increase in the right pleural effusion. There are also small left-sided pleural collections.

Qualitative radiologist review demonstrated that surface-level fluency can coexist with clinically consequential errors, including generic phrasing, missed or overcalled findings, and laterality mismatches. Automated text-based metrics are therefore complementary to, rather than replacements for, structured expert accuracy assessments. This pattern—moderate semantic alignment alongside low structural fidelity—indicates that VLMs may produce fluent report text while substantive factual and relational limitations remain.

One caveat should frame everything that follows. Because this study evaluated a single model on a single public dataset, the conclusions drawn here apply to RadVLM in this experimental setting and are not statements about AI-generated radiology reports in general. Where we describe patterns—low structural fidelity, the weak semantic–fidelity link, improved readability—these describe the system and corpus studied; establishing whether they hold more broadly requires multi-model, multi-dataset replication.

### 4.1 Clinical interpretation of low findings-level fidelity

The observed Findings-level fidelity (RadGraph F1 0.251) warrants direct interpretation rather than mitigation. Two points are essential for placing this value in context. First, RadGraph F1 for free-text report generation must be distinguished from the performance of the RadGraph extraction model itself: the underlying entity–relation extractor attains micro-F1 values near 0.73–0.82 on held-out reference reports (6), so the achievable ceiling for a perfectly faithful generated report is well below 1.0 and the metric is inherently conservative. Second, when judged against this realistic ceiling, generation-side RadGraph F1 values in the published literature remain modest: contemporary chest-radiograph systems typically report RadGraph F1 in the approximate range of 0.15–0.49, with RadVLM itself reporting RadGraph F1 of roughly 0.18 on a filtered MIMIC-CXR test set and comparator vision–language models such as CheXagent and MAIRA-2 occupying a similar band (4, 9). The present value of 0.251, obtained on the independent OpenI corpus, is therefore broadly consistent with—and not markedly below—the prevailing state of the art for narrative report generation, indicating that low structural fidelity reflects a field-wide limitation rather than an implementation artefact specific to this evaluation.

This contextualisation does not soften the clinical implication. No validated RadGraph F1 threshold currently defines clinically acceptable structural fidelity, but a value at which roughly three-quarters of the entity–relation content of a generated report fails to match the reference cannot support autonomous or minimally supervised use. On the evidence presented here, the model is unsuitable for unsupervised clinical deployment and is best characterised as a draft-generation aid that requires radiologist verification of every report before it informs care. The practical question for the field is therefore not whether such models are deployable today—they are not, in an autonomous capacity—but what minimum entity–relation fidelity, validated against radiologist error scores and management-relevant outcomes, should be required before even radiologist-supervised drafting is permitted. Establishing such thresholds, alongside clinically weighted error scoring that distinguishes management-altering errors from trivial discrepancies, is a prerequisite for translating these models into practice.

### 4.2 Relation to prior work on metrics and evaluation

These results are consistent with prior report-generation research showing that surface-level similarity metrics are informative but incomplete, requiring complementary structure-aware checks focused on clinical entities, relations, attributes, and negation (9, 20–23). Fluent reports can contain clinically important factual errors better detected through entity–relation analysis than lexical overlap (9, 21, 22, 24, 25). The joint-model finding that BERTScore-F1 retains an independent association with RadGraph F1 while CheXbert cosine similarity contributes little once semantic similarity is controlled supports the interpretation that label-space agreement overlaps substantially with semantic similarity but does not substitute for entity-aware fidelity (9, 20, 23). A lower Flesch–Kincaid grade level in AI-generated Impressions was interpreted as a stylistic characteristic, not evidence of higher clinical quality, because clinically necessary modifiers may be omitted to achieve simplification (9, 21, 24–28).

Placing these results alongside recent report-generation studies clarifies both the agreement and the differences. Evaluations of general large language models—such as the practical assessment of ChatGPT for radiology report generation (Academic Radiology, 2024)—have found that these systems can produce readable, plausibly structured reports yet still make clinically material errors that lexical overlap metrics fail to catch, echoing our finding that fluency and factual fidelity dissociate. Multi-reader observer studies of AI-drafted chest-radiograph reports have similarly reported that a substantial fraction of AI reports require radiologist correction before they are acceptable, consistent with the draft-aid role we propose for RadVLM. Where the present work differs is in method rather than headline message: instead of comparing a single aggregate similarity score, it applies section-aware routing and then quantifies how weakly Impression-level semantic similarity actually predicts Findings-level entity-relation fidelity, and how that prediction decays with report length and varies across fidelity strata. The RadGraph F1 values observed here also fall within the band reported for dedicated radiology VLMs such as CheXagent and MAIRA-2, suggesting the structural-fidelity shortfall is shared across the current generation of systems rather than unique to the model evaluated.

### 4.3 Interpretation of subgroup findings

The attenuation of the BERTScore-F1–RadGraph F1 association with increasing Impression length suggests that longer summaries may drift from clinically accurate content. The heterogeneous pattern across RadGraph F1 quartiles—where semantic overlap can increase without corresponding gains in entity and relation accuracy—mirrors “plausible but wrong” errors described in prior audits (20, 22, 24). These findings support targeted improvements in content control and entity-aware validation over further optimisation of surface or label-space similarity alone (9, 23–25, 29).

### 4.4 Implications for model development

Given the weak alignment between Impression-level semantic similarity and Findings-level entity–relation fidelity, metric improvements in fluency or semantic overlap should not be interpreted as evidence of improved factual correctness. Post-generation entity–relation checking and structured error characterisation are prioritised as evaluation-oriented next steps. Entity-aware training, explicit verbosity control, and length-constrained generation are hypothesis-generating directions requiring direct comparative testing. Prospective workflow evaluation—measuring time to final sign-off, addendum rates, and reviewer workload—is needed to determine whether metric improvements translate into clinical benefit (4, 10, 23, 27, 29–31).

### 4.5 Implications for deployment and settings with constrained resources

Report-generation systems should be integrated with upstream ordering and downstream communication processes so that well-structured Impressions are consistently linked to verifiable Findings (32, 33). In resource-limited settings—where equipment shortages and limited radiology staff heighten the value of consistent automated support—validation using both semantic-similarity and entity–relation metrics is recommended to detect surface-level and structural errors while maintaining clinical safety (34). High fidelity of entities and relations in Findings is proposed as a prerequisite for deployment so that system acceptance is tied to demonstrable factual reliability (35, 36). These findings align with emerging expectations for transparent, end-to-end evaluation pipelines in chest radiograph report generation (37).

### 4.6 Translation into clinical validation and regulatory pipelines

Section-aware evaluation could be built into the validation pathways that precede clinical adoption. In a practical pipeline, a candidate report-generation system would be scored not on a single similarity number but on section-matched metrics: entity-relation fidelity for the Findings, semantic and label-space agreement for the Impression, and explicit, safety-critical checks for laterality and negation. Thresholds on the fidelity metrics—calibrated against radiologist error scores and management-relevant outcomes rather than chosen arbitrarily—could serve as gating criteria, so that a system advances to supervised clinical piloting only once its structural accuracy clears a defined bar. For regulatory evaluation, the same framework offers a transparent, auditable evidence package: per-section performance, a clinically weighted error taxonomy, and prospective workflow measures such as sign-off time and addendum rates. Embedding these components into validation and post-market surveillance would let approval and monitoring rest on demonstrated factual reliability rather than on surface fluency alone.

### 4.7 Limitations

Several limitations apply. First, evaluation was restricted to a single radiology-tuned model (RadVLM) and a single public corpus (OpenI), with section routing based on XML tags and header heuristics; both choices constrain generalisability, since absolute fidelity levels and the strength of the semantic-similarity–fidelity association may differ for other architectures, institutions, and reporting styles, and the present findings should not be extrapolated to report-generation models as a class without multi-model, multi-dataset replication. Second, the retrospective, association-based design does not support causal inference. Third, all chest radiographs were pooled, and errors were not weighted by clinical importance or detection difficulty; the automated metrics and the qualitative taxonomy therefore do not distinguish management-altering errors from trivial discrepancies, and finding-specific, clinically weighted error scoring is needed to quantify safety-relevant performance. Fourth, automated metrics do not fully characterise clinically consequential failure modes (laterality errors, negation inversion, salience omissions), which require expert adjudication. Fifth, the radiologist audit used a single reviewer without blinded multi-reader assessment, inter-rater agreement, or clinician usability and patient-management impact testing; the study therefore evaluates automated metric behaviour more than adjudicated clinical correctness, and multi-reader validation by two to three thoracic radiologists is required to establish clinical reliability. Sixth, perturbation-based robustness testing was not performed. Finally, external validation on an independent corpus and prospective workflow evaluation were not performed, both necessary before clinical integration is contemplated.

## 5 Conclusion

In this retrospective, metric-based evaluation, RadVLM generated Impression text with moderate semantic similarity (BERTScore-F1 0.498) and substantially lower readability grade level than radiologist-authored Impressions, while Findings-level entity–relation fidelity was low (RadGraph F1 0.251)—a value consistent with the prevailing state of the art for narrative report generation yet far below the level required for unsupervised clinical use. Beyond reaffirming that fluency does not equal factual correctness, the specific contribution of this work is to quantify that dissociation within a section-aware framework: semantic similarity and structural fidelity were only weakly associated, this association attenuated as Impressions lengthened and varied across fidelity strata, and the magnitude of the structural-fidelity shortfall was benchmarked against published systems. Semantic similarity should therefore not be treated as a proxy for structural correctness, and metric gains in fluency or label-space agreement should not be read as evidence of improved factual reliability.

Before clinical use is contemplated, validation with radiologist and clinician involvement is required, using structured accuracy and safety assessments and clinically meaningful error taxonomies. Clinical trustworthiness must be established through dedicated stakeholder-led validation rather than inferred from automated metrics alone.

## Data Availability

The original contributions presented in the study are included in the article/Supplementary Material, further inquiries can be directed to the corresponding author.

## References

[1] Brady AP. Error and discrepancy in radiology: inevitable or avoidable? Insights Imaging. (2016) 8(1):171–82. 10.1007/S13244-016-0534-1 27928712 PMC5265198

[2] Brady AP. Radiology reporting—from hemingway to HAL? Insights Imaging. (2018) 9(2):237–46. 10.1007/S13244-018-0596-3 29541954 PMC5893492

[3] Kennedy E Ryan M England A Sarkodie B Khine R McEntee MF. High workload and under-appreciation lead to burnout and low job satisfaction among radiographers. Radiography. (2025) 31(1):231–40. 10.1016/j.radi.2024.11.019 39631271

[4] Yu F Endo M Krishnan R Pan I Tsai A Reis EP. Evaluating progress in automatic chest x-ray radiology report generation. Patterns. (2023) 4(9):100802. 10.1016/j.patter.2023.100802 37720336 PMC10499844

[5] Smit A Jain S Rajpurkar P Pareek A Ng AY Lungren MP. Chexbert: combining automatic labelers and expert annotations for accurate radiology report labeling using BERT. In: EMNLP 2020—2020 Conference on Empirical Methods in Natural Language Processing, Proceedings of the Conference) (2020), 1500–19. 10.18653/v1/2020.emnlp-main.117

[6] Jain S Agrawal A Saporta A Truong SQ Nguyen Duong D Bui T. RadGraph: extracting Clinical Entities and Relations from Radiology Reports v1.0.0 (2021). Available online at: https://physionet.org/content/radgraph/1.0.0/ 10.13026/hm87-5p47 (Accessed April 17, 2026).

[7] Zhang T Kishore V Wu F Weinberger KQ Artzi Y. BERTScore: evaluating text generation with BERT. In: 8th International Conference on Learning Representations, ICLR 2020) (2019).Available online at: https://arxiv.org/pdf/1904.09675 (Accessed April 17, 2026).

[8] Goldberger AL Amaral LA Glass L Hausdorff JM Ivanov PC Mark RG. Physiobank, PhysioToolkit, and PhysioNet: components of a new research resource for complex physiologic signals. Circulation. (2000) 101(23):e215–20. 10.1161/01.CIR.101.23.e215 10851218

[9] Deperrois N Matsuo H Ruipérez-Campillo S Vandenhirtz M Laguna S Ryser A. RadVLM: a Multitask Conversational Vision-Language Model for Radiology (2025). Available online at: https://arxiv.org/pdf/2502.03333 (Accessed April 17, 2026).

[10] Von Elm E Altman DG Egger M Pocock SJ Gøtzsche PC Vandenbroucke JP. The strengthening the reporting of observational studies in epidemiology (STROBE) statement: guidelines for reporting observational studies. PLoS Med. (2007) 4(10):e296. 10.1371/JOURNAL.PMED.0040296 17941714 PMC2020495

[11] International Committee of Medical Journal Editors. International Committee of Medical Journal Editors. Recommendations for the Conduct, Reporting, Editing, and Publication of Scholarly Work in Medical Journals (2024). Available online at: https://www.icmje.org/recommendations/ (Accessed April 18, 2026). 10.12771/emj.2024.e48 PMC12093628 40704003

[12] Google LLC. Google Colaboratory Pro. Mountain View, CA: Google LLC (2025).

[13] Demner-Fushman D Kohli MD Rosenman MB Shooshan SE Rodriguez L Antani S. Preparing a collection of radiology examinations for distribution and retrieval. J Am Med Inform Assoc. (2015) 23(2):304–10. 10.1093/JAMIA/OCV080 26133894 PMC5009925

[14] Vasilev YA Sychev DA Bazhin AV Shulkin IM Vladzymyrskyy AV Golikova AY. Autonomous artificial intelligence for sorting results of preventive radiological examinations of chest organs: medical and economic efficiency. Digital Diagnostics. (2025) 6(1):5–22. 10.17816/DD641703

[15] Kincaid JP Fishburne RP Jr Rogers RL Chissom BS. Derivation of New Readability Formulas (Automated Readability Index, Fog Count and Flesch Reading Ease Formula) for Navy Enlisted Personnel. National Technical Information Service, Springfield, Virginia 22151 (AD-A006 655/5GA, MF $2.25, PC $3.75). (1975).

[16] Benjaminit Y Hochberg Y. Controlling the false discovery rate: a practical and powerful approach to multiple testing. J R Stat Soc Series B Stat Methodol. (1995) 57(1):289–300. 10.1111/J.2517-6161.1995.TB02031.X

[17] Statsmodels Developers. statsmodels 0.14.6 (2025). Available online at: https://www.statsmodels.org/stable/index.html (Accessed April 18, 2026).

[18] Long JS Ervin LH. Using heteroscedasticity consistent standard errors in the linear regression model. Am Stat. (2000) 54(3):217–24. 10.1080/00031305.2000.10474549

[19] SciPy Developers. SciPy (2025). Available online at: https://scipy.org/ (Accessed April 18, 2026).

[20] Van Ginneken B. The potential of artificial intelligence to analyze chest radiographs for signs of COVID-19 pneumonia. Radiology. (2020) 299(1):E214–5. 10.1148/RADIOL.2020204238 33236962 PMC7993240

[21] Guo L Xia L Zheng Q Zheng B Jaeger S Giger ML. Can AI generate diagnostic reports for radiologist approval on CXR images? A multi-reader and multi-case observer performance study. J Xray Sci Technol. (2024) 32(6):1465–80. 10.3233/XST-240051 39422982 PMC11787813

[22] Babar Z Van Laarhoven T Zanzotto FM Marchiori E. Evaluating diagnostic content of AI-generated radiology reports of chest x-rays. Artif Intell Med. (2021) 116:102075. 10.1016/J.ARTMED.2021.102075 34020752

[23] Hua D Nguyen K Petrina N Young N Cho J-G Yap A. Benchmarking the diagnostic test accuracy of certified AI products for screening pulmonary tuberculosis in digital chest radiographs: preliminary evidence from a rapid review and meta-analysis. Int J Med Inform. (2023) 177:105159. 10.1016/J.IJMEDINF.2023.105159 37549498

[24] Kelly BS Judge C Bollard SM Clifford SM Healy GM Aziz A. Radiology artificial intelligence: a systematic review and evaluation of methods (RAISE). Eur Radiol. (2022) 32(11):7998–8007. 10.1007/S00330-022-08784-6 35420305 PMC9668941

[25] Ram S Bodduluri S. Implementation of artificial intelligence–assisted chest x-ray interpretation: it is about time. Ann Am Thorac Soc. (2023) 20(5):641–2. 10.1513/ANNALSATS.202303-195ED 37126001 PMC10174129

[26] De Vries BM Zwezerijnen GJC Burchell GL Van Velden FHP Menke-van der Houven van Oordt CW Boellaard R. Explainable artificial intelligence (XAI) in radiology and nuclear medicine: a literature review. Front Med (Lausanne). (2023) 10:1180773. 10.3389/FMED.2023.1180773/BIBTEX 37250654 PMC10213317

[27] Huang J Neill L Wittbrodt M Melnick D Klug M Thompson M. Generative artificial intelligence for chest radiograph interpretation in the emergency department. JAMA Netw Open. (2023) 6(10):e2336100. 10.1001/JAMANETWORKOPEN.2023.36100 37796505 PMC10556963

[28] Huang J Wittbrodt MT Teague CN Karl E Galal G Thompson M. Efficiency and quality of generative AI–assisted radiograph reporting. JAMA Netw Open. (2025) 8(6):e2513921. 10.1001/JAMANETWORKOPEN.2025.13921 40471579 PMC12142447

[29] Van Leeuwen KG Schalekamp S Rutten MJCM Van Ginneken B De Rooij M. Artificial intelligence in radiology: 100 commercially available products and their scientific evidence. Eur Radiol. (2021) 31(6):3797–804. 10.1007/S00330-021-07892-Z 33856519 PMC8128724

[30] Colquitt J Jordan M Court R Loveman E Parr J Ghosh I. Artificial intelligence software for analysing chest x-ray images to identify suspected lung cancer: an evidence synthesis early value assessment. Health Technol Assess (Rockv). (2024) 28(50):1–75. 10.3310/LKRT4721 PMC11403378 39254229

[31] Han R Acosta JN Shakeri Z Ioannidis JPA Topol EJ Rajpurkar P. Randomised controlled trials evaluating artificial intelligence in clinical practice: a scoping review. Lancet Digit Health. (2024) 6(5):e367–73. 10.1016/S2589-7500(24)00047-5 38670745 PMC11068159

[32] Almanaa M. Impact of computerized physician order entry (CPOE) coupled with clinical decision support (CDS) on radiologic services. Cureus. (2024) 16(9). 10.7759/CUREUS.69470 PMC11479669 39411619

[33] Almanaa M Jabour A Matabi M Alahmad H Alhulail A Alshuhri M. Evaluating MRI and CT scan scheduling workflows: a retrospective analysis. J Radiat Res Appl Sci. (2024) 17(4):101201. 10.1016/J.JRRAS.2024.101201

[34] Hilabi BS Alghamdi SA Almanaa M. Impact of magnetic resonance imaging on healthcare in low- and middle-income countries. Cureus. (2023) 15(4). 10.7759/CUREUS.37698 37081900 PMC10112545

[35] Almanaa M. Trends and public perception of artificial intelligence in medical imaging: a social Media analysis. Cureus. (2024) 16(9). 10.7759/CUREUS.70008 PMC11498353 39445247

[36] Jung HK Kim K Park JE Kim N. Image-Based generative artificial intelligence in radiology: comprehensive updates. Korean J Radiol. (2024) 25(11):959–81. 10.3348/KJR.2024.0392 39473088 PMC11524689

[37] Yang Y You X Zhang K Fu Z Wang X Ding J. Spatio-Temporal and retrieval-augmented modeling for chest x-ray report generation. IEEE Trans Med Imaging. (2025) 44(7):2892–905. 10.1109/TMI.2025.3554498 40131745

