# Supplementary material for: Bridging semantics and clinical fidelity: a section-based assessment of a vision–language model (RadVLM) for chest x-ray report generation
Source: Front Digit Health. 2026 Aug 27;8:1882716. doi: 10.3389/fdgth.2026.1882716 (PMC13558191; doi:10.3389/fdgth.2026.1882716)
Supplement: Supplementary file 1 [file Supplementaryfile1.docx]

# Supplementary S1: Prompts, Decoding Configuration, and Section Routing

This document provides the exact prompt templates and key generation settings used for RadVLM report generation, together with a concise description of the section extraction and routing rules used for metric computation.

## A. Prompt templates

A.1 Findings-only prompt (applied with the frontal CXR as the only image input):

You are a professional radiologist. Look only at this single frontal chest X-ray.
Write the Findings section ONLY as 3–6 short sentences using this order and scope:
1) Cardiomediastinum/heart size; 2) Lungs (opacities/Consolidation/Atelectasis/Edema);
3) Pleura (effusions/pneumothorax); 4) Lines/tubes/devices; 5) Bones/soft tissues.
Do NOT include section headers or prompt words. Do NOT mention priors, demographics, or dates.
Avoid hedging and speculation; state only what is visible in this image. Output only the findings text.

A.2 Impression-only prompt (applied using the model-generated Findings text as input):

You are a professional radiologist. Based ONLY on the Findings below, write the Impression.
Summarize and synthesize the Findings WITHOUT adding any new information.
1–3 sentences maximum. Do NOT include any section headers or prompt words.
If the findings are overall normal, respond with: 'No acute cardiopulmonary abnormality.'
Findings:
{findings}

Output only the impression text.

## B. Decoding and reproducibility settings

- Decoding strategy: deterministic decoding (do_sample = False; greedy decoding with default beam settings).
- Maximum generated length: max_new_tokens = 240.
- Repetition controls: no_repeat_ngram_size = 3; repetition_penalty = 1.05.
- Temperature sampling: not used (temperature = None).
- Randomness control: random, NumPy, and torch seeds fixed (SEED = 0); cuDNN deterministic mode enabled.

## C. Section extraction and routing

Reference report sections were extracted from Open-I XML when section tags were present; when sections were not explicitly tagged, canonical section headers (e.g., 'FINDINGS', 'IMPRESSION') and formatting cues were used for inference.

Generated text was stored separately as Findings and Impression, consistent with the two-prompt generation procedure.

Metric routing was implemented as follows: RadGraph F1 was computed using the Findings sections, whereas BERTScore-F1, CheXbert cosine similarity, and readability were computed using the Impression sections.

Cases with missing or empty reference Impressions after section parsing were excluded from paired-Impression analyses (e.g., BERTScore-F1) but were retained for analyses that did not require paired Impressions.
