# Supplementary material for: Bridging semantics and clinical fidelity: a section-based assessment of a vision–language model (RadVLM) for chest x-ray report generation
Source: Front Digit Health. 2026 Aug 27;8:1882716. doi: 10.3389/fdgth.2026.1882716 (PMC13558191; doi:10.3389/fdgth.2026.1882716)
Supplement: Supplementary file 2 [file Supplementaryfile2.docx]

# Supplementary S2. STROBE Checklist (Cross-Sectional Study)

This checklist was completed to document alignment with the STROBE reporting recommendations for observational studies.

| **Item** | **Recommendation** | **Location in manuscript** |
| --- | --- | --- |
| 1 | Indicate study design in title/abstract; provide balanced summary. | Title; Abstract |
| 2 | Explain scientific background and rationale. | 1. Introduction |
| 3 | State specific objectives and/or prespecified hypotheses. | 1. Introduction |
| 4 | Present key elements of study design early in the paper. | 2.1. Study design and overview |
| 5 | Describe setting, locations, and relevant dates (including periods of data collection). | 2.2. Data source and preparation |
| 6a | Describe eligibility criteria and sources/methods of participant (case) selection. | 2.2. Data source and preparation |
| 6b | For cross-sectional studies: describe methods of case ascertainment, if applicable. | 2.2. Data source and preparation |
| 7 | Clearly define outcomes, exposures, predictors, potential confounders, and effect modifiers. | 2.5. Metrics and operationalization |
| 8 | For each variable of interest, describe data sources and assessment methods; address comparability. | 2.2; 2.5 |
| 9 | Describe efforts to address potential sources of bias. | 2.3. Model and rationale for selection; 4.5. Limitations |
| 10 | Explain how the study size was determined. | 2.2. Data source and preparation |
| 11 | Explain handling of quantitative variables (e.g., groupings and rationale). | 2.5; 2.6 |
| 12a | Describe statistical methods, including those used to control for confounding. | 2.6. Statistical analysis |
| 12b | Describe methods for subgroup and interaction analyses. | 2.6. Statistical analysis; 3.6–3.7. Subgroup analyses |
| 12c | Explain how missing data were addressed. | 2.6. Statistical analysis (as applicable) |
| 12d | Describe analytical methods that account for sampling strategy (if relevant). | Not applicable (no sampling weights or complex survey design) |
| 12e | Describe sensitivity analyses. | 3.5. Sensitivity analysis; 2.6 |
| 13a | Report numbers of individuals at each stage of the study (e.g., eligible, included, analyzed). | 2.2; 3. Results |
| 13b | Give reasons for non-participation at each stage. | Not applicable (publicly available dataset; pre-defined evaluation set) |
| 13c | Consider use of a flow diagram. | Not included (dataset selection was straightforward); can be added if required |
| 14a | Give characteristics of study participants and information on exposures/confounders. | 2.2; 3. Results |
| 14b | Indicate number of participants with missing data for each variable of interest. | 2.6 (as applicable) |
| 15 | Report outcome events or summary measures. | 3.1–3.7 |
| 16a | Give unadjusted estimates and, if applicable, confounder-adjusted estimates with precision. | 3.3. Relationships among metrics; 3.4. Generalized linear models (GLMs) |
| 16b | Report category boundaries when continuous variables were categorized. | 2.5 (as applicable) |
| 16c | If relevant, translate estimates into absolute risk over a meaningful time period. | Not applicable (cross-sectional, metric-based evaluation) |
| 17 | Report other analyses performed (e.g., subgroup analyses and sensitivity analyses). | 3.5–3.8 |
| 18 | Summarize key results with reference to study objectives. | 4. Discussion; 5. Conclusion |
| 19 | Discuss study limitations, considering direction and magnitude of potential bias or imprecision. | 4.5. Limitations |
| 20 | Provide cautious overall interpretation considering objectives, limitations, and other evidence. | 4. Discussion |
| 21 | Discuss generalizability (external validity) of the study results. | 4.4; 4.5 |
| 22 | Provide source of funding and role of funders. | Funding |
