# Supplementary material for: Bridging semantics and clinical fidelity: a section-based assessment of a vision–language model (RadVLM) for chest x-ray report generation
Source: Front Digit Health. 2026 Aug 27;8:1882716. doi: 10.3389/fdgth.2026.1882716 (PMC13558191; doi:10.3389/fdgth.2026.1882716)
Supplement: Supplementary file 3 [file Supplementaryfile3.docx]

# Supplementary S3. Goodness-of-fit and sensitivity analyses for the association models

Link-function adequacy for the fractional logit (GLM) models was assessed using binned calibration of observed versus predicted RadGraph F1 across deciles of predicted values (Supplementary Figure S3-1). As a distributional sensitivity analysis, beta regression with a logit mean link was fitted after applying a standard epsilon adjustment that mapped RadGraph F1 values into the open interval (0,1). Standardized coefficient estimates (per 1 SD increase in each predictor) and corresponding p-values are summarized in Supplementary Table S3-1.

Supplementary Figure S3-1. Fractional logit (GLM) calibration plot (deciles).

Supplementary Table S3-1. Comparison of fractional logit (GLM) and beta regression specifications.

| **Model** | **Predictor set** | **N** | **BERTScore-F1 coef (p)** | **CheXbert cosine coef (p)** | **Notes** |
| --- | --- | --- | --- | --- | --- |
| Fractional logit (GLM, HC3) | BERTScore-F1 only | 2977 | 0.1535 (<0.001) | — | Predictors z-standardized. |
| Fractional logit (GLM, HC3) | CheXbert cosine only | 3000 | — | 0.1143 (<0.001) | Predictors z-standardized. |
| Fractional logit (GLM, HC3) | BERTScore-F1 + CheXbert cosine | 2977 | 0.1663 (<0.001) | −0.0191 (0.390) | Predictors z-standardized. |
| Beta regression (logit mean link) | BERTScore-F1 only | 2977 | 0.1701 (<0.001) | — | Outcome epsilon-adjusted to (0,1). |
| Beta regression (logit mean link) | CheXbert cosine only | 3000 | — | 0.0595 (0.037) | Outcome epsilon-adjusted to (0,1). |
| Beta regression (logit mean link) | BERTScore-F1 + CheXbert cosine | 2977 | 0.2894 (<0.001) | −0.1750 (<0.001) | Outcome epsilon-adjusted to (0,1). |

**A case-level negative-control (shuffled pairing) artifact intended for sanity checking of RadGraph scoring behavior has been described in Supplementary S4.**
