# Supplementary material for: Bridging semantics and clinical fidelity: a section-based assessment of a vision–language model (RadVLM) for chest x-ray report generation
Source: Front Digit Health. 2026 Aug 27;8:1882716. doi: 10.3389/fdgth.2026.1882716 (PMC13558191; doi:10.3389/fdgth.2026.1882716)
Supplement: Supplementary file 4 [file Supplementaryfile4.docx]

**Supplementary S4. Confusion-style summary of entity-level matching in RadGraph evaluation (Findings section).**

Counts summarize matched entities (true positives), spurious entities (false positives), and missed entities (false negatives) aggregated across the 3,000 cases. Derived precision/recall/F1 were computed from aggregate counts.

| **Quantity** | **Value** |
| --- | --- |
| Cases (n) | 3,000 |
| Ground-truth entities (Σ) | 41,282 |
| AI-predicted entities (Σ) | 40,855 |
| True positives (Σ) | 11,005 |
| False positives (Σ) | 29,850 |
| False negatives (Σ) | 30,277 |
| Precision (TP/(TP+FP)) | 0.269 |
| Recall (TP/(TP+FN)) | 0.267 |
| F1 (from aggregate counts) | 0.268 |

Per-case medians (TP, FP, FN) were 4, 10, and 10, respectively; corresponding medians for ground-truth and AI-predicted entity counts were 15 and 14.

**Negative-control (shuffled pairing) artifact for RadGraph scoring (Findings section).**

To support a reproducible sanity check that entity–relation fidelity is sensitive to correct case-level pairing, a negative-control pairing artifact was generated by randomly permuting AI-generated Findings texts across cases, thereby breaking the correspondence between each study’s reference Findings and the model-generated Findings. The resulting file (radgraph_per_case_shuffled.csv; 3,000 rows) contains case identifiers with the paired reference Findings and mismatched AI-generated Findings. This artifact was retained for optional downstream scoring to verify that RadGraph F1 collapses under incorrect pairing; it was not used for the primary analyses or inference reported in the manuscript. The file is available upon reasonable request.
