# Supplementary material for: Bridging semantics and clinical fidelity: a section-based assessment of a vision–language model (RadVLM) for chest x-ray report generation
Source: Front Digit Health. 2026 Aug 27;8:1882716. doi: 10.3389/fdgth.2026.1882716 (PMC13558191; doi:10.3389/fdgth.2026.1882716)
Supplement: Supplementary file 5 [file Supplementaryfile5.docx]

**Supplementary S5. Key per-case metrics with 95% confidence intervals.**

| **Metric** | **N** | **Mean** | **95% CI (normal) lower** | **95% CI (normal) upper** | **95% CI (bootstrap) lower** | **95% CI (bootstrap) upper** |
| --- | --- | --- | --- | --- | --- | --- |
| RadGraph F1 (Findings) | 3000 | 0.2506 | 0.2446 | 0.2566 | 0.2445 | 0.2566 |
| RadGraph precision (Findings) | 3000 | 0.2701 | 0.2637 | 0.2764 | 0.2637 | 0.2761 |
| RadGraph recall (Findings) | 3000 | 0.2515 | 0.2449 | 0.2581 | 0.2447 | 0.2584 |
| BERTScore-F1 (Impression: paired) | 2977 | 0.4981 | 0.4949 | 0.5012 | 0.4950 | 0.5011 |
| CheXbert cosine similarity (Impression) | 3000 | 0.3942 | 0.3889 | 0.3994 | 0.3894 | 0.3989 |
| Flesch–Kincaid grade (reference Impression) | 3000 | 15.3256 | 15.0155 | 15.6357 | 15.0264 | 15.6270 |
| Flesch–Kincaid grade (AI Impression) | 3000 | 11.7238 | 11.5058 | 11.9418 | 11.5149 | 11.9394 |
| Flesch–Kincaid grade difference (AI – reference: paired) | 2977 | -3.7514 | -4.1180 | -3.3849 | -4.1057 | -3.3650 |
