# Supplementary material for: Bridging semantics and clinical fidelity: a section-based assessment of a vision–language model (RadVLM) for chest x-ray report generation
Source: Front Digit Health. 2026 Aug 27;8:1882716. doi: 10.3389/fdgth.2026.1882716 (PMC13558191; doi:10.3389/fdgth.2026.1882716)
Supplement: Supplementary file 6 [file Supplementaryfile6.docx]

**Supplementary S6. Pairwise correlations among primary metrics with 95% confidence intervals.**

Confidence intervals were derived using percentile bootstrap resampling (B = 1,000; seed = 20251024).

| **Metric pair** | **N** | **Pearson r (95% CI)** | **Spearman ρ (95% CI)** | **Kendall τ (95% CI)** |
| --- | --- | --- | --- | --- |
| RadGraph F1 vs BERTScore-F1 | 2977 | 0.178 (0.140, 0.210) | 0.114 (0.076, 0.148) | 0.076 (0.049, 0.099) |
| RadGraph F1 vs CheXbert cosine | 3000 | 0.128 (0.094, 0.165) | 0.115 (0.080, 0.150) | 0.078 (0.055, 0.103) |
| BERTScore-F1 vs CheXbert cosine | 2977 | 0.670 (0.651, 0.690) | 0.671 (0.651, 0.691) | 0.479 (0.463, 0.495) |
